# 7-Ketocholesterol Induces Lipid Metabolic Reprogramming and Enhances Cholesterol Ester Accumulation in Cardiac Cells

**DOI:** 10.3390/cells10123597

**Published:** 2021-12-20

**Authors:** Mei-Ling Cheng, Hsiang-Yu Tang, Pei-Ting Wu, Cheng-Hung Yang, Chi-Jen Lo, Jui-Fen Lin, Hung-Yao Ho

**Affiliations:** 1 Metabolomics Core Laboratory, Healthy Aging Research Center, Chang Gung University, Taoyuan City 33302, Taiwan; chengm@mail.cgu.edu.tw (M.-L.C.); tangshyu@mail.cgu.edu.tw (H.-Y.T.); blackismystyle@gmail.com (C.-H.Y.); chijenlo@mail.cgu.edu.tw (C.-J.L.); rflin@mail.cgu.edu.tw (J.-F.L.); 2 Clinical Metabolomics Core Laboratory, Chang Gung Memorial Hospital, Taoyuan City 33305, Taiwan; 3 Graduate Institute of Biomedical Sciences, College of Medicine, Chang Gung University, Taoyuan City 33302, Taiwan; teresaloveyoyo@gmail.com; 4 Department of Biomedical Sciences, College of Medicine, Chang Gung University, Taoyuan City 33302, Taiwan; 5 Department of Medical Biotechnology and Laboratory Science, College of Medicine, Chang Gung University, Taoyuan City 33302, Taiwan; 6 Research Center for Emerging Viral Infections, Chang Gung University, Taoyuan 33302, Taiwan

**Keywords:** 7-ketocholesterol, cholesteryl esters, cardiac cells

## Abstract

7-Ketocholesterol (7KCh) is a major oxidized cholesterol product abundant in lipoprotein deposits and atherosclerotic plaques. Our previous study has shown that 7KCh accumulates in erythrocytes of heart failure patients, and further investigation centered on how 7KCh may affect metabolism in cardiomyocytes. We applied metabolomics to study the metabolic changes in cardiac cell line HL-1 after treatment with 7KCh. Mevalonic acid (MVA) pathway-derived metabolites, such as farnesyl-pyrophosphate and geranylgeranyl-pyrophosphate, phospholipids, and triacylglycerols levels significantly declined, while the levels of lysophospholipids, such as lysophosphatidylcholines (lysoPCs) and lysophosphatidylethanolamines (lysoPEs), considerably increased in 7KCh-treated cells. Furthermore, the cholesterol content showed no significant change, but the production of cholesteryl esters was enhanced in the treated cells. To explore the possible mechanisms, we applied mRNA-sequencing (mRNA-seq) to study genes differentially expressed in 7KCh-treated cells. The transcriptomic analysis revealed that genes involved in lipid metabolic processes, including MVA biosynthesis and cholesterol transport and esterification, were differentially expressed in treated cells. Integrated analysis of both metabolomic and transcriptomic data suggests that 7KCh induces cholesteryl ester accumulation and reprogramming of lipid metabolism through altered transcription of such genes as sterol O-acyltransferase- and phospholipase A2-encoding genes. The 7KCh-induced reprogramming of lipid metabolism in cardiac cells may be implicated in the pathogenesis of cardiovascular diseases.

## 1. Introduction

The oxidized low-density lipoprotein (LDL) in the artery wall is known to participate in atherogenesis [1,2]. A cytotoxic component of oxidized low-density lipoproteins—7-Ketocholesterol (7KCh)—is believed to contribute to the atherosclerotic process. High levels of 7KCh are detected in advanced atherosclerotic plaques [3] and in the plasma of patients with increased risk for cardiovascular diseases [4,5]. Although 7KCh can be catabolized in the liver [6,7], extrahepatic metabolism of 7KCh acts through its esterification to fatty acids by cytosolic sterol O-acyltransferase (SOAT) and subsequent selective efflux to high-density lipoprotein (HDL) [8]. Lowered expression of SOAT1 and SOAT2 in heart tissue [8] may lead to an accumulation of 7KCh and exacerbate heart damage in patients with cardiovascular disease. Our recent findings indicate that 7KCh is highly enriched in the red blood cells of patients with heart failure, and this result implies that 7KCh may act as an early risk factor for heart failure [9]. Additionally, we also demonstrate that 7KCh promotes reactive oxygen species (ROS) formation and then induces growth inhibition or death in cardiomyocytes [9].

Although 7KCh is consistently cytotoxic to cells, its physiological effect on cardiomyocytes is unknown. Oxysterols can inhibit the mevalonic acid (MVA) pathway and cholesterol (Chol) biosynthesis [3,10]. The MVA pathway uses acetyl-CoA, NADPH, and ATP to produce sterols and isoprenoids and plays a key role in a variety of biological processes [11]. Initially, the regulation and function of the MVA pathway and its metabolites were studied in the context of normal and hypercholesterolemic tissues [11,12]. In recent years, the importance of MVA pathway-derived metabolites in different fields, such as cancer and immune systems [13,14,15,16], has become increasingly appreciated. In cardiac cells, inhibition of the MVA pathway prevents anoxia- or ischemia-induced cardiac dysfunction [17,18]. The MVA route is involved in regulating the growth of cardiomyocytes [19,20]; however, the effect of 7KCh on the MVA pathway and its subsequent effects on cardiomyocytic metabolism and growth are seldom investigated.

The heart uses a plethora of substrates to meet energetic demands for continual contraction. Because fatty acids are the predominant fuel for the adult heart, the regulation of lipid metabolism plays a vital role in cardiac cells. Herein, we integrated the transcriptomic data and metabolic profiles to delineate the effect of 7KCh on metabolism, including the MVA pathway, in cardiac cells. The transcriptomic data revealed that the triacylglycerol (TG) pathway- and Chol biosynthetic pathway-related genes are downregulated, while SOAT and phospholipase A2 (PLA2) are upregulated in 7KCh-treated cardiomyocytes. The present study offers insight into the effect of 7KCh on lipid metabolism in cardiac cells. 

## 2. Materials and Methods

### 2.1. Materials

Unless otherwise stated, all chemicals were purchased from Sigma-Aldrich (St. Louis, MO, USA). The Claycomb medium and norepinephrine for the cultivation of HL-1 cells were purchased from Sigma-Aldrich. The HL-1 qualified fetal bovine serum, penicillin/streptomycin, and glutamine were purchased from EMD Millipore (Burlington, MA, USA), and 7-Ketocholesterol (7KCh; C2394; available from Sigma-Aldrich) was dissolved in dimethyl sulfoxide (DMSO). The stock solution was further diluted in a culture medium for use.

### 2.2. Cell Culture and Growth Curve Determination

HL-1 atrial myocytes (Research Resource Identifier (RRID): CVCL_0303; Sigma-Aldrich Catalogue No: SCC065) were acquired from Sigma-Aldrich and were cultured as previously described [21]. To determine the growth curves of untreated control (i.e., treated with vehicle; Con) and 7KCh-treated cells, 5 × 10^4^ cells were seeded in a 12-well culture plate and incubated with vehicle (diluted DMSO) or with different concentrations of 7KCh for the indicated periods. The 7KCh concentrations used in the present study corresponded to those encountered under pathophysiological conditions. It is estimated that the blood 7KCh levels of healthy volunteers range from 1 to 2 μM and are elevated 10–20 fold in heart failure (HF) patients [9]. Cardiac cells were fixed in 3.7% formaldehyde and stained with 5 μg/mL Hoechst 33342. The cell number was determined using IN Cell Analyzer 1000 (GE Healthcare Life Sciences, Chicago, IL, USA) [22]. 

### 2.3. Global Metabolite Analysis by Ultrahigh-Performance Liquid Chromatography Time-of-Flight Mass Spectrometry (UPLC-TOF-MS)

Extraction was carried out as previously described [23,24]. In brief, the medium was removed from the culture plates, and 80% methanol (prechilled at −80 °C) was immediately added. Cells were scraped from the culture dish. The resulting cell suspension was vortexed and centrifuged at 14,000× *g* for 15 min. It was re-extracted once more with 80% methanol at −80 °C. The samples were pooled and dried under nitrogen gas. They were then dissolved in 200 μL 50% acetonitrile, and the supernatant was analyzed using UPLC-TOF-MS. 

An ACQUITY BEH Amide (2.1 mm × 150 mm, particle size: 1.7 μm) (Waters Corp., Milford, MA, USA) column was used for liquid chromatographic separation. Separation was maintained at a flow rate of 400 μL/min and a temperature of 45 °C. A linear gradient of solvents was used: 0–0.1 min, 1% B; 0.1–7.0 min, 1–70% B; 7.0–7.2 min, 1% B, and 7.2–10.0 min, 1% B for re-equilibration. Solvent A was acetonitrile, solvent B was water, and both solvents contained 0.1% formic acid. Each sample was analyzed in triplicate. Mass spectrometric analysis was performed using a Waters SYNAPT G2-S HDMS TOF-MS (Waters Corp.) operated in the positive or negative ion mode. The desolvation gas flow was set at 800 L/h at a temperature of 500 °C, and the source temperature was 120 °C. The capillary voltage and cone voltage were adjusted to 2 kV and 25 V, respectively. 

All data were analyzed using Progenesis QI software (Nonlinear Dynamics, Newcastle, UK). The identities of metabolites were revealed by searching METLIN [25] and Human Metabolome databases [26] or by spectral comparison with standard compounds. 

### 2.4. Quantification of Coenzymes and Sterols by Liquid Chromatography Coupled with Tandem Mass Spectrometry (LC-MS/MS)

Intracellular reduced and oxidized coenzymes and sterols were extracted using a modified method [27]. Briefly stated, the culture medium was removed, and the cells were extracted with 1-propanol containing internal standards (Chol-d6, 7KCh-d7, coenzyme Q10-d9 (CoQ10-d9)) for analysis of Chol, 7KCh and coenzyme Qs (CoQs). For CoQ determination, samples were analyzed using UPLC coupled with Waters Xevo TQ-S MS (Waters Corp.) as previously described with slight modifications [27]. For Chol and 7KCh determination, samples were analyzed using UPLC coupled with Waters Xevo TQ-S MS (Waters Corp., Milford, MA, USA) according to a modified method [28]. MS was performed in ESI-positive ion multiple reaction monitoring (MRM) mode. The MS parameters were as follows: the cone gas was 150 L/h, the capillary voltage was 1.1 kV, the desolvation temperature was 500 °C, the desolvation gas flow was 1000 L/h, and the source temperature was 150 °C. Chromatographic separation was achieved on a BEH C18 column (100 mm × 2.1 mm; particle size: 1.7 μm; Waters Corp.) at 60°C with an isogradient mobile phase (5 mM ammonium formate in methanol with 0.05% formic acid). 

### 2.5. Quantification of Dolichols in HL-1 Cell by UPLC-TOF-MS

Cells were suspended in PBS and extracted with chloroform/methanol/PBS (1/2/0.8) using a modified method described by Bligh and Dyer [29]. After 15 min incubation at room temperature, the sample was subjected to centrifugation, and the supernatant was retained. Chloroform and PBS were added to form a mixture of chloroform, methanol, and PBS (2:2:1.8). The sample was centrifuged once more. The lower phase was collected and dried under nitrogen gas. 

For dolichol determination, samples were resuspended in methanol and analyzed using UPLC coupled with Waters Xevo G2-XS TOF MS. A BEH C8 column was used for separation at 50 °C. The mobile phase consisted of solvent A (methanol: acetonitrile: 1 mM ammonium acetate; 60:20:20, *v*/*v*/*v*) and solvent B (1 mM ammonium acetate in ethanol). The sample was eluted at a flow rate of 0.2 mL/min, and the gradient elution was performed as follows: 0–6 min, 30–99% B; 99% B for an additional 2 min. The source temperature was 120 °C; the desolvation temperature was 500 °C; the capillary voltage was 2 kV in negative ion mode; the cone voltage was 40 V, and the desolvation gas was 800 L/h. 

### 2.6. Quantification of Metabolites in MVA Pathway by LC-MS/MS

Cells were scraped in methanol and processed as described in Section 2.3. The sample, dried under nitrogen gas, was dissolved in 100 μL 50% methanol for analysis.

Analysis of metabolites in the MVA pathway was achieved using a UPLC coupled with Waters Xevo TQ-S MS according to a modified method described by Sugimoto et al. [30]. A BEH C18 column was employed for metabolite separation at 30 °C. The mobile phase consisted of solvent A (10 mM ammonium carbonate containing 0.1% ammonium hydroxide) and solvent B (acetonitrile/methanol (75:25, *v*/*v*) containing 0.1% ammonium hydroxide). The flow rate was set at 0.25 mL/min, and the gradient elution was performed as follows: 10% B, 0.5 min; 10–65% B, 6 min; 65% B, 2 min; 65–95% B, 0.5 min; 95% B, 2 min. MS analysis was conducted using a tandem MS in negative ion mode. The following MS condition was used: The capillary voltage was set at 0.5 kV. The desolvation gas flow rate was 1000 L/h, and the cone gas flow was set at 150 L/h. The desolvation and source temperatures were 500 °C and 150 °C, respectively.

### 2.7. Quantification of Cholesteryl Esters (CE) and Triglycerides by LC-MS/MS

Cells were extracted with 1-propanol and collected in a microtube. The sample was centrifuged, and the supernatant was dried with nitrogen gas. The sample was resuspended in IPA/acetonitrile/water (2:1:1, *v*/*v*/*v*), and the supernatant was retained for cholesteryl ester and triglyceride analysis. 

Cholesteryl esters were separated on a BEH C18 column and detected with Xevo TQ-S MS (Waters Corp.) operated in positive-ion mode. The column temperature was set at 60 °C. The mobile phase consisted of solvent A (acetonitrile/water (40:60, *v*/*v*) with 10 mM ammonium formate), and the solvent B (isopropanol/acetonitrile (90:10, *v*/*v*) with 10 mM ammonium formate). Elution was achieved at a flow rate of 0.45 mL/min, and the gradient elution was performed as follows: 0–10 min, 40–99% solvent B, and 10–10.1 min, 99–40% solvent B. The capillary voltage was 1 kV, the cone voltage was 30 V, the desolvation gas flow was 1000 L/h, and the cone gas flow was 150 L/h. The desolvation and source temperatures were respectively adjusted to 500 °C and 150 °C. Triglycerides were separated on a Waters CORTECS T3 column (2.1 mm × 30 mm × 2.7 µm) (Waters Corp) and detected using Xevo TQ-S MS operated in positive-ion mode. The column was maintained at a temperature of 60 °C. The mobile phase consisted of solvent A (0.01% formic acid in water) and solvent B (isopropanol/acetonitrile (50:50, *v*/*v*) containing 0.01% formic acid). Elution was achieved at a flow rate of 0.25 mL/min, and the gradient elution was performed as follows: 90% B, 2 min; 90–98% B, 4 min; and 98% B, 2 min. The following MS condition was used: The cone gas flow was maintained at 150 L/h. The cone voltage was 35 V, and the capillary voltage was 3.5 V. The desolvation gas flow rate was 1000 L/h, and the desolvation and source temperatures were adjusted to 500 °C and 150 °C, respectively.

### 2.8. mRNA-seq Profiling and Data Analysis

The mRNA-seq profiling was performed by Novogene. Sequencing libraries were constructed with the NEBNext UltraTM RNA Library Prep Kit, and sequencing was conducted on an Illumina NovaSeq platform. The paired-end reads were generated, and the filtered reads were aligned with the sequences from the Genome Reference Consortium Mouse Build 38 using the TopHat2 tool. The normalized count values were output by the fragments per kilobase per million (FPKM) method. Network Analyst 3.0 [17] delineated the biological pathways and processes and the protein–metabolite interactions.

### 2.9. Statistical Analyses

The principal component analysis (PCA) and orthogonal partial least squares discriminate analysis (OPLS-DA) of the MS data were performed using the SIMCA-P+ vs. 13.0 (Umetrics, Umeå, Sweden). The variable importance in the projection (VIP) scores for metabolites were computed. Results are the mean ± SD (for continuous variable). All statistical analyses were accomplished using IBM SPSS 20.0 (Armonk, NY, USA) and R Version 4.0.2 (R Development Core Team; https://www.r-project.org/). The Student’s *t*-test was used to compare data. FDR corrections were used for data comparison where appropriate. A *p* value of <0.05 was considered statistically significant. 

## 3. Results

### 3.1. Intracellular 7KCh Accumulation Is Associated with Cell Growth Inhibition 

**Figure 1:**
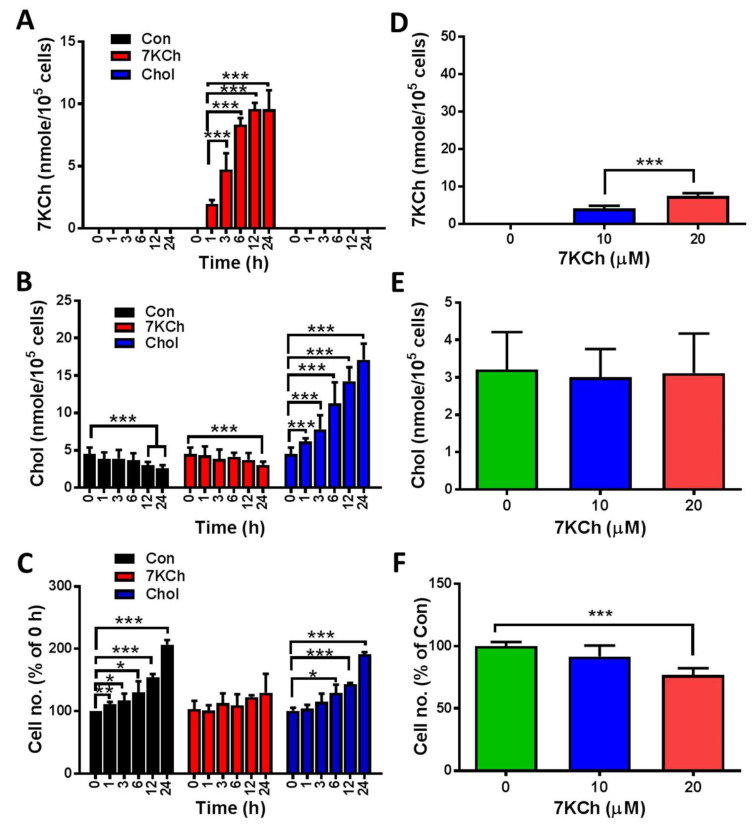
Growth inhibition and 7KCh accumulation in HL-1 cells. (**A**–**C**) HL-1 cells (5 × 10^4^/well) were treated without (Con) or with 20 μM 7KCh (or Chol) for indicated periods (0, 1, 3, 6, 12, and 24 h). These cells were harvested for LC-MS-based quantification of the intracellular 7KCh (**A**) and Chol (**B**). Data are mean ± SD of three experiments. The numbers of the cells treated for different periods were determined and expressed as the percentage of that of the untreated HL-1 cells (**C**). Data are mean ± SD of three experiments. * *p* < 0.05, ** *p* < 0.01, *** *p* < 0.005, vs. Con. (**D**–**F**) Cells were treated with different concentrations (0, 10, and 20 μM) of 7KCh for 24 h and then harvested for quantification of intracellular 7KCh (**D**) and Chol (**E**). Data are mean ± SD of six experiments. *** *p* < 0.005, vs. Con. The numbers of the cells treated with different 7KCh concentrations were determined and are expressed as the percentage of that of untreated HL-1 cells (**F**). Data are mean ± SD of six experiments. *** *p* < 0.005, vs. Con.

To examine whether 7KCh impairs the physiology of cardiomyocytes, we tested the changes in the growth of HL-1 cells after 7KCh treatments. The levels of 7KCh in healthy volunteers ranged from 1 to 2 μM [31], but the blood levels of 7KCh in heart failure patients were at least 10- to 20-fold higher than those of the normal controls [9]. The concentration of 7KCh used for cell treatment ranged from 10 μM to 20 μM. 7KCh caused a dose-dependent reduction in cell number at concentrations ranging from 10 μM to 20 μM in HL-1 cells at 24 h post-treatment (Figure 1F). To follow the time-dependent change in the growth of HL-1 cells, we treated HL-1 cells with 20 μM 7KCh for different periods (0, 1, 3, 6, 12, and 24 h) and determined the cell number (Figure 1C). The number of vehicle control-treated cells increased nearly 2-fold 24 h later, while the number of 7KCh-treated cells increased slightly, suggesting that 7KCh causes growth retardation in HL-1 cells. To test whether 7KCh treatment causes its intracellular accumulation, we applied LC-MS/MS to determine the intracellular levels of Chol and 7KCh in HL-1 cells. 7KCh accumulated intracellularly in a dose- and time-dependent manner (Figure 1A,D). However, the intracellular Chol levels did not change significantly in cells treated with 20 μM 7KCh throughout the 24-h period and those treated with 7KCh concentrations up to 20 μM (Figure 1B,E). On the contrary, the Chol treatment that caused intracellular Chol accumulation (Figure 1B) but not that of 7KCh (Figure 1A) did not affect the cell growth (Figure 1C).

### 3.2. The Metabolic Profiles of 7KCh-Treated HL-1 Cells

**Figure 2:**
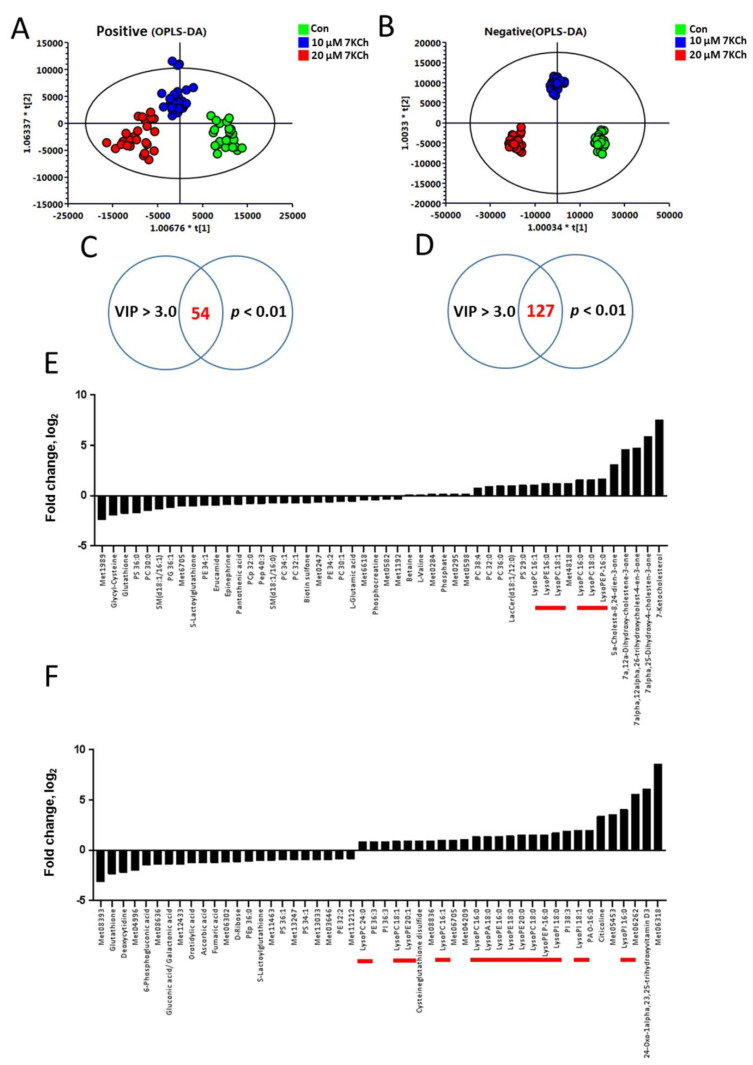
Changes in global metabolism in 7KCh-treated HL-1 cells. For global metabolomic analysis, HL-1 cells were untreated (Con) or treated with the indicated concentrations (10, 20 μM) of 7KCh for 24 h, and harvested for subsequent analyses by LC-MS and LC-MS/MS in ESI-positive (**A**,**C**,**E**) and ESI-negative (**B**,**D**,**F**) modes. The corresponding OPLS-DA score plots are shown (**A**,**B**). Significantly different metabolites between 7KCh-treated and control cells were selected with VIP score > 3 and *p* value < 0.01. The log_2_ fold changes in the abundance of metabolites differentially abundant in cells treated with 20 μM 7KCh versus Con cells (i.e., the log_2_ of the ratio of the abundance of a metabolite in 7KCh group to that of Con group) are plotted (**E**,**F**). The lysophospholipids are highlighted by the red bars.

To study the changes in global metabolism of cardiomyocytes in response to 7KCh, we treated HL-1 cells without (i.e., vehicle-treated) or with different concentrations of 7KCh, and then analyzed the metabolome using LC-TOF-MS. Typical spectra of HL-1 cell extract were obtained in positive and negative ion modes. After data processing with Progenesis QI, the data were analyzed with SIMCA-P. The OPLS-DA score plots are shown in Figure 2. The score plots showed that the metabolite profiles of HL-1 cells treated with different 7KCh concentrations were spatially separated in both ESI positive and negative modes (Figure 2A,B). The metabolite profiles of HL-1 changed significantly in a dose-dependent manner after 7KCh treatment. Fifty-four and 127 metabolites acquired in ESI positive and negative modes were selected based on the criteria of variable importance in the projection (VIP) scores > 3.0 and significant differences (*p* < 0.01) between the untreated and those treated with 20 μM 7KCh (Figure 2C,D). The top 50 significantly changed metabolites are presented in Figure 2E,F. These findings suggest that sterols, phospholipids, and lysophospholipids are important discriminators of untreated cells and those treated with 7KCh. The levels of lysophospholipids (such as lysophosphatidylcholines (lysoPCs) and lysophosphatidylethanolamines (lysoPEs)), and oxysterols were elevated, while the levels of many phospholipids decreased in 7KCh-treated cells compared with those of the untreated cells. Furthermore, glutathione decreased in 7KCh-treated cells.

### 3.3. 7KCh Affects Transcription of Lipid Metabolism-Related Genes

**Figure 3:**
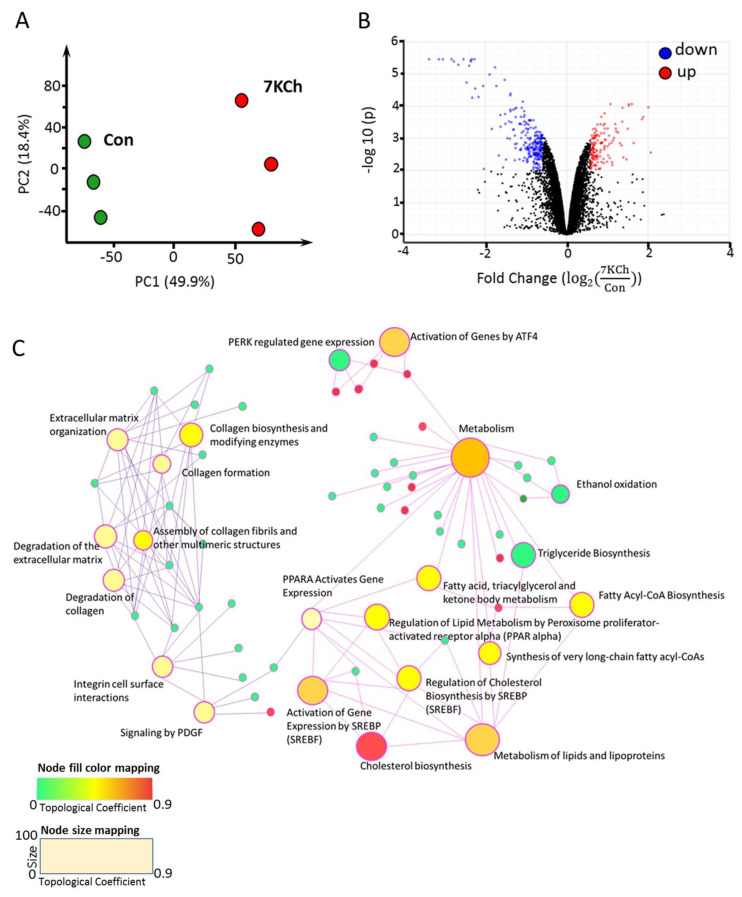
mRNA-seq profiling of 7KCh-treated cardiac cells. (**A**) HL-1 cells were untreated (Con) or treated with 20 μM 7KCh for 24 h and harvested for mRNA-seq analysis by Novogene. The principal component analysis (PCA) plot of the mRNA-seq data from control (green) and 7KCh treatment (red) groups is shown. (**B**) The volcano plot highlights the upregulated (up, red) and downregulated (down, blue) genes in 7KCh-treated cells (versus Con cells). The fold change ($\log_{2}\left(\frac{7\mathrm{KCh}}{\mathrm{Con}}\right)$) is indicative of $\log_{2}\left(\frac{\mathrm{Expression}\;\mathrm{units}\;\left(\mathrm{in}\;\mathrm{FPKM}\right)\;\mathrm{of}\;\mathrm{the}\;\mathrm{gene}\;\mathrm{in}\;7\mathrm{KCh}-\mathrm{treated}\;\mathrm{cells}}{\mathrm{Expression}\;\mathrm{units}\;\left(\mathrm{in}\;\mathrm{FPKM}\right)\;\mathrm{of}\;\mathrm{the}\;\mathrm{gene}\;\mathrm{in}\;\mathrm{control}\;\left(\mathrm{Con}\right)\mathrm{cells}}\right)$ for the specified gene (*n* = 3, q value < 0.001). (**C**) Gene association network and module crosstalk network of all significantly altered genes are shown. The default network was generated by Network Analyst. The size of the nodes is based on their degree values, with big size for large degree values. The color of nodes is proportional to their betweenness centrality values.

The PCA plot revealed significant changes in transcriptomes of control and 7KCh-treated cells (Figure 3A). Expression of 401 genes changed significantly in 7KCh-treated HL-1 cells versus untreated control (adjusted *p*-value < 0.01 and fold change > 1.5). These genes included 257 upregulated and 144 downregulated genes. These differentially expressed genes (DEGs) are presented by a volcano plot (Figure 3B). Data mapping to KEGG and Reactome revealed several significantly altered pathway gene sets. These pathway gene sets included those implicated in Chol biosynthesis, sterol regulatory element-binding transcription factor (SREBF)-mediated gene activation, SREBF-regulated Chol biosynthesis, metabolism of lipids and lipoproteins, and ATF4-mediated gene activation (Figure 3C). These enriched pathways with differentially upregulated and downregulated genes are summarized in Table 1. The gene sets associated with ATF4-mediated gene activation, PERK regulated-pathway, and unfolded protein response pathway were upregulated (Figure 3C and Table 1) after 24 h-treatment of HL-1 cells with 20 μM 7KCh.

### 3.4. Alteration in Gene Expression and Metabolites Involved in MVA Pathway in 7KCh-Treated Cardiac Cells

**Figure 4:**
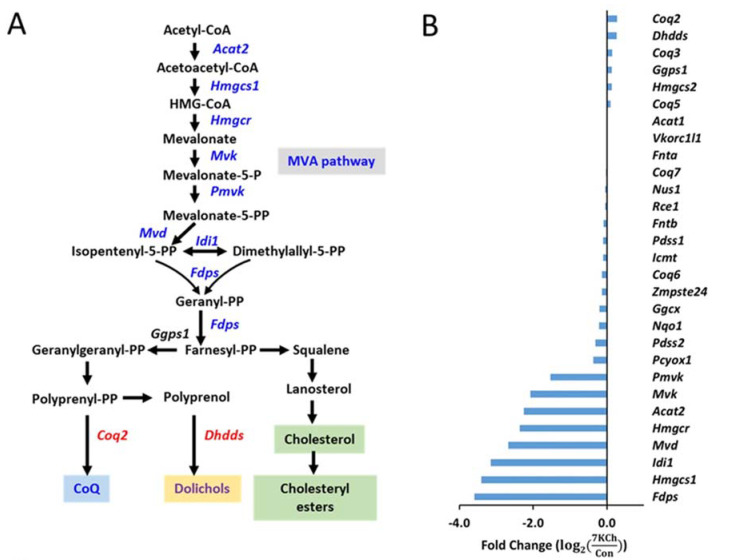

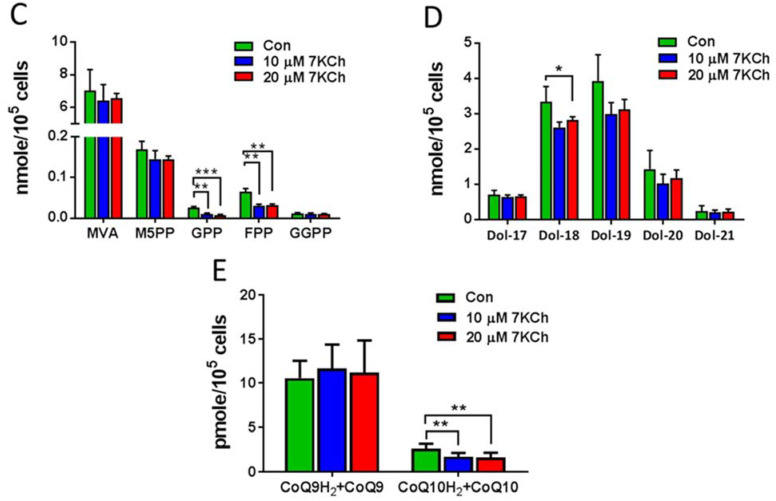
Effect of 7KCh on MVA pathway in cardiac cells. (**A**) A simplified diagram illustrating the mevalonate (MVA) pathway and its conversion to cholesterol, dolichol, coenzyme Q, and farnesyl-PP and geranylgeranyl-PP. Genes marked in blue represent the downregulated genes after 7KCh treatment, while those marked in red represent the upregulated ones. (**B**) Changes in expression levels of MVA pathway-related genes in HL-1 cells treated with 20 μM 7KCh for 24 h are shown as the fold change ($\log_{2}\left(\frac{7\mathrm{KCh}}{\mathrm{Con}}\right)$) that is indicative of $\log_{2}\left(\frac{\mathrm{Expression}\;\mathrm{units}\;\left(\mathrm{in}\;\mathrm{FPKM}\right)\;\mathrm{of}\;\mathrm{the}\;\mathrm{gene}\;\mathrm{in}\;7\mathrm{KCh}-\mathrm{treated}\;\mathrm{cells}}{\mathrm{Expression}\;\mathrm{units}\;\left(\mathrm{in}\;\mathrm{FPKM}\right)\;\mathrm{of}\;\mathrm{the}\;\mathrm{gene}\;\mathrm{in}\;\mathrm{control}\;\left(\mathrm{Con}\right)\mathrm{cells}}\right)$ for the specified gene. The differentially changed genes include *Hmgcs1*, *Idi1*, *Hmgcr*, *Mvk*, *Acat2*, *Mvd*, *Pmvk*, *Fdps*, *Pcyox1*, *Coq2* and *Dhdds* (adjusted *p* value < 0.05, *n* = 3). The accession number and descriptions of these genes are given in Supplementary Table S1. (**C**–**E**) HL-1 cells were untreated (*Control*) or treated with indicated concentrations of 7KCh for 24 h and harvested for LC-MS/MS-based determination of intermediates in the MVA pathway (**C**), dolichols (**D**), and CoQs (**E**). Data are mean ± SD of three experiments. * *p* < 0.05, ** *p* < 0.01, *** *p* < 0.001. Mevalonate, MVA; mevalonate-5-pyrophosphate, M5PP; geranyl pyrophosphate, GPP; farnesyl pyrophosphate, FPP; geranylgeranyl pyrophosphate, GGPP; Dolichol, Dol; coenzyme Q, CoQ.

**Figure 5:**
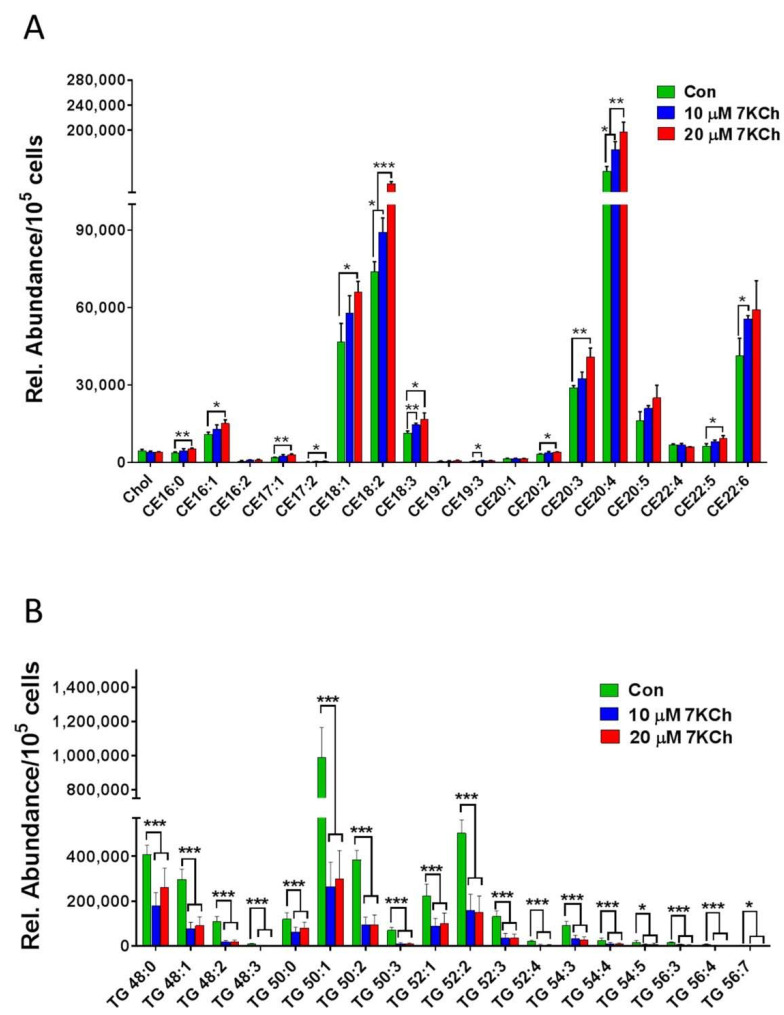
7KCh alters intracellular levels of TGs and CEs in HL-1 cells. HL-1 cells were untreated (Con) or treated with indicated concentrations (10 or 20 μM) of 7KCh for 24 h and harvested for LC-MS/MS-based quantification of TGs (**A**) and CEs (**B**). The relative abundance (Rel. Abundance) of each metabolite per 10^5^ cells is shown. Data are mean ± SD of three experiments. * *p* < 0.05, ** *p* < 0.01, *** *p* < 0.001.

Of the 7KCh-induced transcriptomic changes in HL-1 cells, the expression of a number of genes was altered. These genes encode enzymes involved in the MVA pathway and the subsequent conversion reactions, such as *Fdps, Hmgcs1, Idi1, Mvd, Hmgcr,* *Acat2, Mvk, Pmvk, Pcyox1, Dhdds*, and *Coq2* (Figure 4A). There were decreases in expression of the MVA pathway-associated genes in 7KCh-treated cells, compared to those of control (Con) cells (Figure 4B). Downregulation of these genes may affect the biosynthesis of Chol (Figure 4C), dolichol (Figure 4D), and coenzyme Q (Figure 4E). As expected, the levels of MVA derivatives geranyl pyrophosphate (GPP) and farnesyl-pyrophosphate (FPP) decreased in 7KCh-treated cells, whereas MVA-5-pyrophosphate (M5PP) and geranylgeranyl pyrophosphate (GGPP) levels remained unchanged (Figure 4C). Downstream products dolichols and CoQs are mildly affected by 7KCh. The dolichol-18 level was reduced modestly but significantly, while the dolichol-19 level decreased nonsignificantly. CoQ9 did not change, while the CoQ10 level was significantly reduced in the 7KCh-treated cells (Figure 4D,E). The Chol level did not decrease significantly (Figure 1B,E), while a number of cholesterol esters (CEs) increased in abundance (Figure 5A).

### 3.5. 7KCh Reduces Triacylglyceride Synthesis in Cardiac Cells

Genes involved in free fatty acid and triglyceride biosynthesis were downregulated (Table 1). Consistent with this, the intracellular triglycerides (TGs) in abundance decreased significantly (Figure 5B). These findings suggest that 7KCh inhibits TG biosynthesis.

## 4. Discussion

The blood levels of 7KCh are associated with cardiovascular disease events [4,5,9]. As the enzymes required for 7KCh catabolism are expressed in the liver, 7KCh catabolism in other tissues like the heart and any intervention measures to reduce its toxicity remain to be investigated. This study integrated metabolomic and transcriptomic data to study the 7KCh-induced alteration of metabolic pathways in cardiac cells and represents the first one to delineate how an oxysterol induces lipid metabolic reprograming and enhances CEs accumulation in cardiac cells. 

**Figure 6:**
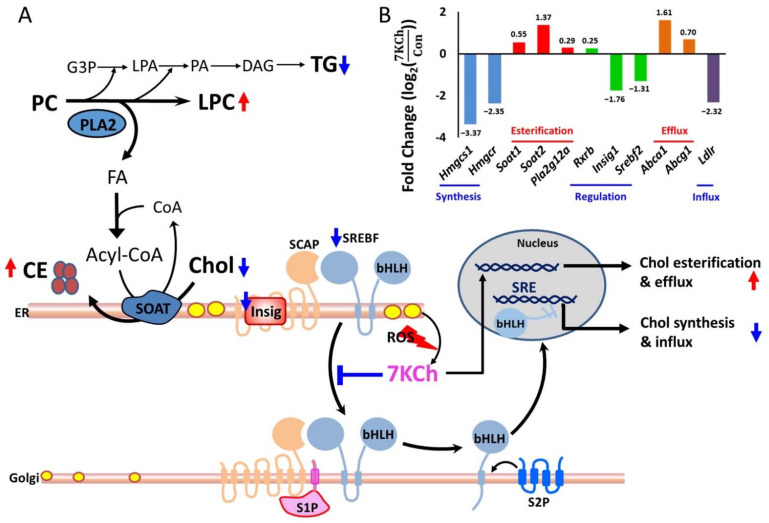
A schematic diagram illustrating 7KCh-induced remodeling of lipid metabolism in HL-1 cells. **(A)** 7KCh inhibits TG biosynthesis and activates PLA2 to release fatty acid (FA) from phospholipids (e.g., PC). This provides FA for the esterification of Chol (yellow oval) to form CE (brown oval). 7KCh inhibits cleavage and activation of SREBF. Decreased expression of *Srebf2* and *Insig1* genes may also reduce the transcriptional activity of SREBF. Inhibition of SREBF-mediated transcription reduces the expression of genes involved in the MVA pathway and biosynthesis. Additionally, 7KCh may activate cell stress response that involves ATF4 and PERK signaling pathways (not shown here). Metabolites marked in blue (or red) represent those metabolites whose levels are lowered (or elevated) in 7KCh-treated cells versus control cells. 7-Ketocholesterol, 7KCh; cholesterol, Chol; cholesterol ester, CE; triglyceride, TG; phosphatidylcholine, PC; lysophosphatidylcholine, lysoPC; glycerol-3-phosphate G3P; lysophosphatidate, LPA; phosphatidate, PA; diacylglyceride, DAG; fatty acid, FA; sterol regulatory element-binding transcription factor, SREBF; SREBF chaperone, SCAP; insulin-induced gene protein, Insig; site-1 protease, S1P; site-2 protease, S2P. (**B**) A significant difference in the expression of genes involved in lipid synthesis between 7KCh-treated cells and control cells is shown. The fold change ($\log_{2}\left(\frac{7\mathrm{KCh}}{\mathrm{Con}}\right)$) is indicative of $\log_{2}\left(\frac{\mathrm{Expression}\;\mathrm{units}\;\left(\mathrm{in}\;\mathrm{FPKM}\right)\;\mathrm{of}\;\mathrm{the}\;\mathrm{gene}\;\mathrm{in}\;7\mathrm{KCh}-\mathrm{treated}\;\mathrm{cells}}{\mathrm{Expression}\;\mathrm{units}\;\left(\mathrm{in}\;\mathrm{FPKM}\right)\;\mathrm{of}\;\mathrm{the}\;\mathrm{gene}\;\mathrm{in}\;\mathrm{control}\;\left(\mathrm{Con}\right)\mathrm{cells}}\right)$ for the specified gene (adjusted *p* value < 0.05, *n* = 3). The accession number and descriptions of these genes are given in Supplementary Table S1. The genes are categorized according to their functions in cholesterol metabolism. It is noted that the *Insig* gene (*Insig1*), *SREBF* gene (*Srebf2*), and cholesterol influx-associated gene low density lipoprotein receptor (*Ldlr*) are downregulated in response to 7KCh.

The enhanced production of cholesteryl esters is probably associated with SREBF- and PPARα-mediated upregulated expression of downstream genes, such as *Soat* genes, in 7KCh-treated cells. Decreased expression of genes involved in fatty acyl-CoA biosynthesis suggests a reduction in fatty acid synthesis. As the fatty acids synthesis declined, fatty acids required for esterification of 7KCh were supplied through the catalytic action of phospholipase A2 (PLA2) in cardiomyocytes. The lysophospholipids were markedly higher in 7KCh-treated cells than untreated cells, which is consistent with the enhanced expression of PLA2G12A (phospholipase A2, group XIIA). The proposed scheme is shown in Figure 6.

The metabolism of 7KCh is noteworthy. The extrahepatic metabolism of 7KCh is mainly through its esterification to fatty acids by cytosolic SOAT and possibly through its subsequent selective efflux to HDL [8]. Phospholipase A2 (PLA2) catalyzes the hydrolysis of membrane glycerophospholipids to liberate free fatty acids. It has been reported that oxidized LDL can activate PLA2 to supply fatty acids for Chol esterification in macrophages [32]. 7KCh induces apoptosis in macrophages by a mechanism involving cPLA2, but it also forms part of a second message when esterified by SOAT [33]. Pharmacological inhibition [34] or knockout [35] of macrophage SOAT results in increased plaque size in animal models of atherosclerosis. An increase in apoptotic cells was observed in the *Soat1*-/- lesions. The failure to metabolize oxidized Chol properly by macrophages may contribute to cytotoxicity and pathogenesis of vascular diseases. The present study demonstrated that 7KCh induces metabolic reprogramming in cardiomyocytes. Such a process is probably implicated in the pathogenesis of cardiovascular diseases. The transcriptomic data showed that the Chol synthesis-related genes and the Chol influx-associated gene, such as *Ldlr* gene, are downregulated, while the Chol efflux and cholesteryl ester synthesis-associated genes, such as *Abca1*, *Abcg1*, *Soat1*, *Soat2*, and *Pla2g12a*, are upregulated in 7KCh-treated cardiomyocytes (Figure 6). ABCA1 and SOAT appear to play a role in reducing the accumulation of free sterol. Increased PLA2 expression and lysoPC accumulation in cardiomyocytes may represent the cellular effort to supply sufficient fatty acids for CE esterification.

Taken together, the transcriptomic and metabolic changes may represent a compensatory cytoprotective response of cardiomyocytes to 7KCh. Moreover, excessive formation of lysophospholipids may have a detrimental effect on cardiomyocytes. Enhanced lysophosphatidylcholine (LPC) production has been associated with apoptotic cardiomyopathy in high-fructose and high-fat-fed animal models [36]. Arachidonyl oxysterol that can be generated by SOAT may act as an apoptotic signal [33]. The relationship involving PLA2, SOAT, and accumulation of lipid metabolites in 7KCh-treated cardiomyocytes may be more complicated than previously thought. It is plausible that the fine-tuned gene regulatory and metabolic networks exist in cardiomyocytes to ensure proper cellular responses to oxysterols. The destiny of cells may depend on various inputs to these networks (such as oxysterol level and availability of certain metabolites, e.g., PC) and the presence of regulatory elements (such as expression levels of specified genes, e.g., those encoding SOAT and PLA2).

The inhibitory effect of 7KCh on the MVA pathway in cardiomyocytes has been seldom discussed. The MVA pathway plays a key role in a variety of biological processes [11]. In mammalian cells, the MVA pathway is the intracellular source of isopentenyl pyrophosphate (IPP), the precursor of Chol (steroid biosynthesis), farnesyl-pyrophosphate, and geranylgeranyl-pyrophosphate, dolichol (N-glycan biosynthesis), and coenzyme Q (ubiquinone biosynthesis) [11]. Chol is an integral component of cellular membranes, the precursor of steroid hormones, vitamin D, and bile acids [11,37]. Chol depletion impairs cardiac contraction [38]. Dolichol is an essential component of the N-glycosylation of nascent polypeptides in the ER [39,40]. IPP molecules are used to produce the quinone CoQ. The hydrophobic isoprenoid chain of CoQ is used to localize the mitochondrial inner membrane, where the quinone group serves as an electron carrier and enables ATP production [41,42]. Reduction in expression of MVA pathway-related genes and the intermediates GPP and FPP suggests a decrease in the flux of the MVA pathway. However, the downstream products such as CoQ and dolichols are only mildly affected. It may be accounted for by upregulation of *Coq2* and *Dhdds* gene expression, which may represent a compensatory mechanism to maintain CoQ and dolichols under the condition of the reduced flux of the MVA pathway.

Cardiac TG homeostasis is mainly determined by the balance between de novo synthesis, endogenous TG catabolism, and probably the re-esterification of DAGs originating from TG breakdown. The heart has an extremely high turnover rate of TGs and contributes to mitochondrial FA supply [43]. The de novo pathway of TG formation is initiated by glycerol-3-phosphate acyltransferases (GPATs)-catalyzed FA esterification at the *sn*-1 position of glycerol-3-phosphate (G3P), resulting in the formation of lysophosphatidic acid (LPA) [12]. Fatty acyl moieties can be incorporated into phospholipids (e.g., PC) by lysophospholipid acyltransferases or released from them by PLA2 [44]. Two pathways involved in PC metabolism, the Kennedy pathway and Lands cycle, act coordinately to regulate triacylglycerol content. The metabolomic results show that the 7KCh-treated cardiac cells have reduced TG content. The transcriptomic results reveal the downregulation of the expression of TG biosynthesis-related genes. As cardiac TG formation and lipolysis determine the amount of FAs that can be used for energy metabolism, defective cardiac TG homeostasis may result in metabolic aberration, lipotoxicity, and cardiac dysfunction. Apart from the increases in PLA2 activity and lipolysis, FAs can be made available for cholesteryl ester formation by reducing de novo TG biosynthesis. Decreases in both TGs and phospholipids may adversely affect the physiology of cardiomyocytes. The proposed scheme is shown in Figure 6. The present study gives an insight into the 7KCh-induced reprogramming of lipid metabolism in cardiac cells.

## Figures and Tables

**Table 1:** Pathways with differentially upregulated and downregulated genes.

Pathway	Total	Expected	Hits	*p* Value	FDR
Upregulated genes in 7KCh-treated cardiac cell					
Activation of Genes by ATF4	7	0.047	4	6.12 × 10^−8^	8.02 × 10^−5^
PERK regulated gene expression	10	0.0671	4	3.62 × 10^−7^	2.37 × 10^−4^
Unfolded Protein Response	66	0.443	6	4.38 × 10^−6^	1.91 × 10^−3^
Circadian Clock	39	0.262	4	0.000123	3.22 × 10^−2^
PPARA Activates Gene Expression	78	0.523	5	0.00016	3.22 × 10^−2^
Amino acid synthesis and interconversion (transamination)	21	0.141	3	0.000346	3.48 × 10^−2^
Downregulated genes in 7KCh-treated cardiac cell					
Cholesterol biosynthesis	28	0.449	18	1.35 × 10^−26^	1.77 × 10^−23^
Activation of Gene Expression by SREBP (SREBF)	32	0.513	15	2.04 × 10^−19^	1.33 × 10^−16^
Regulation of Cholesterol Biosynthesis by SREBP (SREBF)	49	0.786	16	1.36 × 10^−17^	5.94 × 10^−15^
Metabolism of lipids and lipoproteins	553	8.87	37	1.08 × 10^−14^	3.54 × 10^−12^
Metabolism	1600	25.6	55	7.37 × 10^−10^	1.93 × 10^−7^
Fatty Acyl-CoA Biosynthesis	18	0.289	6	2.36 × 10^−7^	5.16 × 10^−5^
Collagen biosynthesis and modifying enzymes	52	0.834	8	1.42 × 10^−6^	2.56 × 10^−4^
Triglyceride Biosynthesis	37	0.594	7	1.57 × 10^−6^	2.56 × 10^−4^
Extracellular matrix organization	159	2.55	12	7.90 × 10^−6^	1.00 × 10^−3^
Assembly of collagen fibrils and other multimeric structures	47	0.754	7	8.38 × 10^−6^	1.00 × 10^−3^
Fatty acid, triacylglycerol, and ketone body metabolism	160	2.57	12	8.43 × 10^−6^	1.00 × 10^−3^
Collagen formation	73	1.17	8	1.93 × 10^−5^	2.10 × 10^−3^
Degradation of collagen	54	0.867	7	2.15 × 10^−5^	2.17 × 10^−3^
Integrin cell surface interactions	86	1.38	8	6.39 × 10^−5^	5.98 × 10^−3^
Degradation of the extracellular matrix	87	1.4	8	6.95 × 10^−5^	6.07 × 10^−3^
Ethanol oxidation	8	0.128	3	0.000212	1.74 × 10^−2^

## Data Availability

The data presented in this study are available on request from the corresponding author. The data are not publicly available due to patent application request.

## Supplementary Materials

The following are available online at https://www.mdpi.com/article/10.3390/cells10123597/s1, Table S1: gene list in Figure 4 and Figure 6.
